# Continuous presence of genetically diverse rustrela virus lineages in yellow-necked field mouse reservoir populations in northeastern Germany

**DOI:** 10.1093/ve/vead048

**Published:** 2023-07-28

**Authors:** Sina Nippert, Dennis Rubbenstroth, Jessica Anna Geers, Arnt Ebinger, Donata Hoffmann, Angele Breithaupt, Claudia Wylezich, Xuejing Wang, Viola C Haring, Philip Starcky, Paola Fruci, Christoph Langner, Christin Trapp, Heiko Schulz, Wilko Stubbe, Christian Imholt, Gerald Heckel, Martin Beer, Florian Pfaff, Rainer G Ulrich

**Affiliations:** Institute of Novel and Emerging Infectious Diseases, Friedrich-Loeffler-Institut, Federal Research Institute for Animal Health, Südufer 10, Greifswald-Insel Riems 17493, Germany; Institute of Novel and Emerging Infectious Diseases, Friedrich-Loeffler-Institut, Federal Research Institute for Animal Health, Südufer 10, Greifswald-Insel Riems 17493, Germany; Institute of Diagnostic Virology, Friedrich-Loeffler-Institut, Federal Research Institute for Animal Health, Südufer 10, Greifswald-Insel Riems 17493, Germany; Institute of Diagnostic Virology, Friedrich-Loeffler-Institut, Federal Research Institute for Animal Health, Südufer 10, Greifswald-Insel Riems 17493, Germany; Institute of Diagnostic Virology, Friedrich-Loeffler-Institut, Federal Research Institute for Animal Health, Südufer 10, Greifswald-Insel Riems 17493, Germany; Department of Experimental Animal Facilities and Biorisk Management, Friedrich-Loeffler-Institut, Federal Research Institute for Animal Health, Südufer 10, Greifswald-Insel Riems 17493, Germany; Institute of Diagnostic Virology, Friedrich-Loeffler-Institut, Federal Research Institute for Animal Health, Südufer 10, Greifswald-Insel Riems 17493, Germany; Department of Experimental Animal Facilities and Biorisk Management, Friedrich-Loeffler-Institut, Federal Research Institute for Animal Health, Südufer 10, Greifswald-Insel Riems 17493, Germany; Institute of Ecology and Evolution, University of Bern, Baltzerstraße 6, Bern CH-3012, Switzerland; Institute of Novel and Emerging Infectious Diseases, Friedrich-Loeffler-Institut, Federal Research Institute for Animal Health, Südufer 10, Greifswald-Insel Riems 17493, Germany; Institute of Diagnostic Virology, Friedrich-Loeffler-Institut, Federal Research Institute for Animal Health, Südufer 10, Greifswald-Insel Riems 17493, Germany; Institute of Diagnostic Virology, Friedrich-Loeffler-Institut, Federal Research Institute for Animal Health, Südufer 10, Greifswald-Insel Riems 17493, Germany; Laboratory of Infectious Diseases, Faculty of Veterinary Medicine, University of Teramo, Via Renato Balzarini 1, Teramo 64100, Italy; Stralsund Zoological Garden, Grünhufer Bogen 2, Stralsund 18437, Germany; Tierpark Grimmen, Friedrichstraße 20, Grimmen 18507, Germany; Betriebsteil Forstplanung/Versuchswesen/Informationssysteme, Landesforst Mecklenburg-Vorpommern—Anstalt des öffentlichen Rechts, Zeppelinstraße 3, Schwerin 19061, Germany; Institut für Allgemeine und Systematische Zoologie, Universität Greifswald, Loitzer Straße 26, Greifswald 17489, Germany; Rodent Research, Institute for Epidemiology and Pathogen Diagnostics, Julius Kühn-Institute (JKI), Federal Research Centre for Cultivated Plants, Toppheideweg 88, Münster 48161, Germany; Institute of Ecology and Evolution, University of Bern, Baltzerstraße 6, Bern CH-3012, Switzerland; Institute of Diagnostic Virology, Friedrich-Loeffler-Institut, Federal Research Institute for Animal Health, Südufer 10, Greifswald-Insel Riems 17493, Germany; Institute of Diagnostic Virology, Friedrich-Loeffler-Institut, Federal Research Institute for Animal Health, Südufer 10, Greifswald-Insel Riems 17493, Germany; Partner Site Hamburg-Lübeck-Borstel-Riems, German Center for Infection Research (DZIF), Germany

**Keywords:** *Matonaviridae*, reservoir host, range, sequence variation, *Apodemus flavicollis*, yellow-necked field mouse

## Abstract

Rustrela virus (RusV; species *Rubivirus strelense*, family *Matonaviridae*) was discovered in different zoo animal species affected by fatal encephalitis. Simultaneous RusV RNA detection in multiple yellow-necked field mice (*Apodemus flavicollis*) suggested this rodent as a reservoir of RusV. Here, we investigated 1,264 yellow-necked field mice and sympatric other small mammals from different regions in Germany for RusV RNA using an optimized reverse transcription-quantitative polymerase chain reaction (RT-qPCR) protocol and high-throughput sequencing. The investigation resulted in the detection of RusV RNA exclusively in 50 of 396 (12.6 per cent) yellow-necked field mice but absence in other sympatric species. RT-qPCR-determined tissue distribution of RusV RNA revealed the highest viral loads in the central nervous system, with other tissues being only very rarely affected. The histopathological evaluation did not reveal any hints of encephalitis in the brains of infected animals despite the detection of viral RNA in neurons by *in situ* hybridization (ISH). The positive association between the body mass of yellow-necked field mice and RusV RNA detection suggests a persistent infection. Phylogenetic analysis of partial E1 and full-genome sequences showed a high diversification with at least four RusV lineages (1A–1D) in northeastern Germany. Moreover, phylogenetic and isolation-by-distance analyses indicated evolutionary processes of RusV mostly in local reservoir populations. A comparison of complete genome sequences from all detected RusV lineages demonstrated a high level of amino acid and nucleotide sequence variability within a part of the p150 peptide of the non-structural polyprotein and its coding sequence, respectively. The location of this region within the RusV genome and its genetic properties were comparable to the hypervariable region of the rubella virus. The broad range of detected RusV spillover hosts in combination with its geographical distribution in northeastern Germany requires the assessment of its zoonotic potential and further analysis of encephalitis cases in mammals. Future studies have to prove a putative co-evolution scenario for RusV in the yellow-necked field mouse reservoir.

## 1. Introduction

For a long time, rubella virus (RuV; species *Rubivirus rubellae*) represented the only member of the family *Matonaviridae* (Chen et al. 2018). RuV usually causes an exanthematous disease in humans, known as rubella or ‘German measles’, and is endemic in most parts of the world with humans being the only natural reservoir (Knipe and Howley 2007). In 2020, its first relatives rustrela virus (RusV; *Rubivirus strelense*) and ruhugu virus (RuhV; *Rubivirus ruteetense*) were discovered using high-throughput sequencing (HTS) (Bennett et al. 2020a,b; Mankertz et al. 2022; Pfaff et al. 2022). Currently, isolates are available neither for RuhV nor for RusV; thus, the biological properties of these viruses are largely unknown. The single-stranded, non-segmented RNA(+) genome of RusV is about 9,631 nucleotides (nt) long and encodes two polyproteins: (1) the non-structural polyprotein (nsPP) at the 5ʹ end of the genome that is cleaved into the protease p150 (1,093 amino acid [aa] residues) and the RNA-directed RNA polymerase p90 (828 aa); (2) the structural polyprotein (sPP) that is cleaved into the capsid (C) protein (332 aa) and the envelope glycoproteins E2 (324 aa) and E1 (487 aa) (Oker-Blom 1984; Oker-Blom et al. 1984; Oker-Blom, Jarvis, and Summers 1990; Pfaff et al. 2022). The open reading frames for nsPP and sPP are separated by an intergenic region of 290 nt (Pfaff et al. 2022). The high guanine and cytosine content (GC) content of the RusV genome of roughly 70 mol per cent with peaks up to 82 mol per cent caused problems in the initial determination of the complete genome sequence (Bennett et al. 2020b; Das, Kielian, and Parrish 2021; Pfaff et al. 2022). However, a target enrichment-based HTS procedure resulted in the determination of several high-quality genome sequences of RusV from putative reservoir and diseased spillover-infected animals (Pfaff et al. 2022).

RusV has been identified in samples from diverse mammals that suffered from severe encephalitis (Bennett et al. 2020a,b; Pfaff et al. 2022). In detail, RusV was discovered during investigations of a series of three encephalomyelitis cases in a donkey (*Equus asinus*), a capybara (*Hydrochoerus hydrochaeris*), and red-necked or Bennett’s wallaby (*Macropus rufogriseus*) in 2018 and 2019 in a zoological garden in northeastern Germany (Bennett et al. 2020a,b). The detection of three additional RusV-associated encephalomyelitis cases in the zoo (a red-necked wallaby and a South American coati, *Nasua nasua*) and in the surroundings of the zoo (Eurasian or European otter, *Lutra lutra*) suggested an ongoing circulation of RusV in this region (Pfaff et al. 2022; Voss et al. 2022). Furthermore, two cases of RusV-associated encephalomyelitis in red-necked wallabies in additional zoos in northeastern Germany indicated the virus to be more widespread than initially thought (Voss et al. 2022). Rodent pest management in the initially affected zoological garden resulted in the identification of a putative reservoir host, the yellow-necked field mouse (*Apodemus flavicollis*) (Bennett et al. 2020b).

In our study, we aimed to (1) investigate the host specificity of RusV, (2) investigate the range of RusV distribution in yellow-necked field mice and other potentially susceptible rodent species from Germany, (3) characterize individual and population-based factors influencing the RusV prevalence, and (4) evaluate the phylogenetic relationships and sequence evolution of RusV strains.

## 2. Materials and methods

### 2.1 Collection and dissection of small mammals

**Figure 1.**
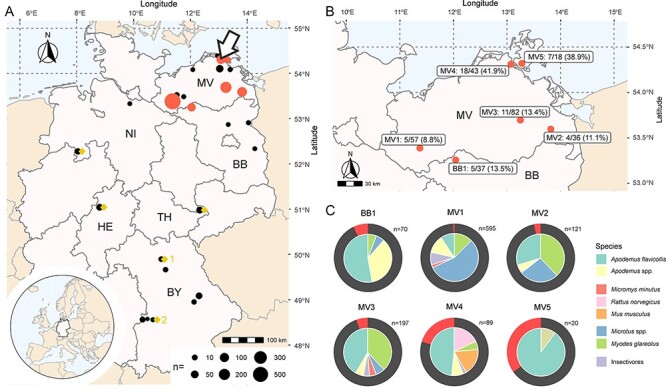
The spatial distribution of RusV detection in Germany. (A) Small mammals were collected at trapping sites in six federal states of Germany. The size of each dot corresponds to the number of trapped and analyzed animals at this location. Trapping sites with at least one RusV-positive animal, as tested by RusV-specific RT-qPCR, are highlighted in red. The location of the initial discovery of RusV in a zoo in northeastern Germany is marked with an arrow. Trapping sites from which RNA pools were analyzed using metagenomic sequencing are highlighted with a yellow diamond (compare Supplementary Table S2). (B) A detailed map with trapping sites MV1-MV5 and BB1 where RusV was solely detected in yellow-necked field mice (*Apodemus flavicollis*). Numbers of trapped yellow-necked field mice and RusV-positive animals are depicted for each trapping site. (C) The species composition of the small mammal populations at trapping sites MV1–MV5 and BB1 is depicted using circle charts. Each section of the inner circles corresponds to the frequency of different species and the outer circle highlights the fraction of RusV-positive animals among all tested individuals using red for RusV-positive or black for RusV-negative animals.

Small mammals were collected during rodent monitoring in parts of Germany by several forestry offices (Drewes et al. 2017), State Office for Consumer Protection and Food Safety, Lower Saxony (Weber de Melo et al. 2015), pest rodent control programs of different zoos, cat trappings, and a biodiversity study on the isle of Rügen (Stubbe 2012) (Fig. 1A). Carcasses were frozen after trapping and shipped on dry ice to the Friedrich-Loeffler-Institut. In the frozen state, the heads were removed and the brains extracted. Half of the brain was used for nucleic acid extraction and further molecular biological investigation. The other half was fixed for potential subsequent histopathological evaluation (see Section 2.13). The rest of each carcass was stored at −20°C for subsequent investigations. A previously described collection of nineteen yellow-necked field mice, six striped field mice, three bank voles, thirteen Norway rats, and sixteen house mice from the initially affected zoological garden and its surroundings (maximum 10 km distance) (Supplementary Table S1) was included in this study (Bennett et al. 2020b; Pfaff et al. 2022).

### 2.2 Nucleic acid extraction

RNA extraction followed a standard protocol using the NucleoMag VET kit (Macherey-Nagel, Düren, Germany). *In vitro*–transcribed RNA of the enhanced green fluorescence protein gene (termed internal control 2 [IC-2]) was supplemented during the extraction process and served as nucleic acid extraction control (Hoffmann et al. 2006). Briefly, a maximum of approximately 100 mg native organ material was added to a 2 ml tube with 500 µl of a mixture of equal volumes of Eagle Minimum Essential Medium (MEM) (Hanks’ balanced salts solution) and Eagle MEM (Earle’s balanced salts solution) supplemented with 2 mM L-glutamine, nonessential amino acids adjusted to 850 mg/l NaHCO_3_, 120 mg/l sodium pyruvate, pH 7.2, and a 5-mm steel bead, followed by mechanical fragmentation for 2 min at 30 Hz using a TissueLyser (Qiagen, Hilden, Germany). Thereafter, 100 µl homogenate was mixed with 100 µl lysis buffer (VL, NucleoMag VET Kit, Macherey-Nagel), 20 µl proteinase K, and 10 µl IC-2 RNA and incubated for 5 min at room temperature. Subsequently, 350 µl binding buffer (VEB) and 20 µl NucleoMag B beads were added. In three steps with two washing buffers (VEW1 and VEW2) and 80 per cent ethanol, nucleic acids were extracted and purified using King Fisher 96 Flex workstation (Thermo Fisher Scientific, Darmstadt, Germany).

### 2.3 Molecular species determination

Morphological species identification was confirmed for selected carcasses by cytochrome *b* (cyt *b*)–specific polymerase chain reaction (PCR), sequence determination, and comparison to GenBank entries (Schlegel et al. 2012).

### 2.4 Screening real-time reverse transcription PCR

The initial RusV RNA screening was performed using a previously established reverse transcription- quantitative polymerase chain reaction (RT-qPCR) protocol (‘Assay 1’; Bennett et al. 2020b, Supplementary Table S2). Based on RusV sequences generated during this study, this RT-qPCR was optimized for the detection of a broader range of RusV variants by replacing the forward primer and probe with the modified versions RusV_1072_A+ and RusV_1116_A_P, respectively (Supplementary Table S2). The reaction mix using the SensiFAST Probe No-ROX One-Step Kit (Meridian Bioscience, Cincinnati, OH, USA) contained final concentrations of 1.5 mg/ml bovine serum albumin, 0.2 µM forward primer, 0.6 µM reverse primer, 0.3 µM TaqMan probe, and 4 µl template RNA in a total volume of 20 µl. Both assays were performed with the following cycler setup: 45°C for 10 min, 95°C for 10 min, 45 cycles of 95°C for 5 sec, and 58°C for 20 sec on a Bio-Rad CFX96 qPCR cycler (Bio-Rad, Feldkirchen, Germany). The cycle of quantification (Cq) value was used for a comparison of viral RNA load. Cq values were standardized using a diluted RNA preparation of a RusV-infected donkey brain (GenBank accession number MN552442.2) with a known Cq value as a positive control in each RT-qPCR run.

### 2.5 Amplification and dideoxy-chain termination sequencing of the E1-encoding region

Partial E1-encoding sequences were amplified by two conventional RT-PCR assays using primer pairs RusV-E1_8188+/RusV-E1_8663- and RusV-E1_8528+/RusV-E1_8941- (final concentration 0.4 μM each; for sequences, see Supplementary Table S2) and the Superscript III One-Step RT-PCR System with Platinum Taq DNA Polymerase (Invitrogen, Carlsbad, CA, USA). After a single step at 50°C for 30 min and 95°C for 2 min, in both assays, 45 cycles of denaturation (94°C for 5 sec) and annealing and elongation (62°C and 57°C, respectively, for 30 sec) with a final step of 5 min incubation at 68°C followed. RT-PCR products of the expected length were excised from 2 per cent agarose gel, purified with Zymoclean Gel DNA Recovery Kit (Zymo Research, Freiburg, Germany), and eluted in 35 μl diethyl pyrocarbonate–treated and sterile filtered water. Dideoxy-chain termination sequencing was performed by Microsynth Seqlab (Göttingen, Germany), with respective forward and reverse primers in both directions. After trimming of primer sequences and stretches of insufficient quality, raw sequences were *de novo* assembled by Geneious Prime (version 2019.2.3) to yield 438 nt or 375 nt for the two individual amplicons and 715 nt for both overlapping sequences combined.

### 2.6 HTS of RusV genomes

Based on the phylogeny of the 715-nt-long sequences, ten RusV-positive samples from different trapping sites were selected for whole-genome sequencing to expand the knowledge of RusV genomic diversity. Sequencing libraries were generated from extracted total RNA and sequenced according to the protocol described in Wylezich et al. (2018). In detail, RNA was extracted from frozen brain tissue of selected RusV-positive animals using the cryoPREP impactor (Covaris, UK) in combination with the RNAdvance Tissue Kit (Beckman Coulter, Germany) on a KingFisher Flex Purification System (Thermo Fisher Scientific). Subsequently, RNA was reverse-transcribed into cDNA using the SuperScript IV First-Strand cDNA Synthesis System (Invitrogen, Germany) and the NEBNext Ultra II Non-Directional RNA Second Strand Synthesis Module (New England Biolabs, Frankfurt am Main, Germany). Ion Torrent–compatible barcoded sequencing libraries were then generated and sequenced on an Ion Torrent S5XL instrument (Thermo Fisher Scientific). When sequencing of the original library did not yield a whole-genome sequence of RusV, target enrichment was applied via the custom ‘panRubi bait set v2’ (Daicel Arbor Biosciences, Ann Arbor, MI, USA) to the sequencing libraries. The design and application of the custom enrichment panel are described elsewhere (Pfaff et al. 2022).

In addition to sequencing individual RusV-positive samples, five pools of brain-derived RNA of yellow-necked field mice and wood mice (*Apodemus sylvaticus*) from Thuringia (TH), Hesse (HE), Lower Saxony (NI), and Bavaria (BY) (Fig. 1A and Supplementary Table S3) were sequenced using the described workflow. In addition, target enrichment using the custom ‘panRubi bait set v3’ was applied to the sequencing libraries. The resulting reads were then checked for the presence of rubiviral genomes using diamond BLASTx (Buchfink, Xie, and Huson 2015) and a custom database with all available sequences within the virus family *Matonaviridae*.

### 2.7 RusV genome assembly

Ion Torrent–derived raw data were trimmed with respect to quality and adapter contamination using 454 Sequencing Systems Software (version 3.0). Reads were then filtered using a GC content cutoff of ≥60 mol per cent in PRINSEQ-lite (version 0.20.4) and were *de novo* assembled with SPAdes (Bankevich et al. 2012) (*sc*, *iontorrent*; version 3.15.2). Contigs representing RusV genomes were quality-checked by back-mapping of trimmed reads and annotated with respect to RusV reference sequence MN552442.2 using Geneious Prime (version 2021.0.1).

### 2.8 Rapid amplification of cDNA ends of the 5ʹ end of the RusV genomes

In order to sequence the 5ʹ end of RusV genomes, cDNA was generated from total RNA using SuperScript III reverse transcriptase (Invitrogen) and 5ʹ Rapid Amplification of cDNA Ends (RACE) 2.0 system (Invitrogen) using a custom protocol. In detail, 1–5 µg of total RNA in 20 µl was combined with 5 µl of primer RusV_323- (1 µM; Supplementary Table S2), heated at 70 ºC for 10 min, and immediately transferred to 50 ºC. In a separated tube, a reaction mix containing PCR buffer (10×; 5 µl), MgCl_2_ (25 mM; 5 µl), deoxyribonucleotide triphosphates (dNTPs) (10 mM; 2.5 µl), dithiothreitol (0.1 M; 5 µl), and SuperScript III reverse transcriptase (200 U; 1 µl) were prepared; subsequently combined with the RNA-primer-mix; and incubated for 50 min at 50°C. After inactivation at 70°C for 10 min, RNase Cocktail (Invitrogen) was added and incubated at 37 °C for 30 min. The cDNA was then cleaned using 1.0× volume of AMPure XP magnetic beads (Beckman Coulter). Poly(A) or poly(C) tails were added to the 3ʹ end of the cleaned cDNA using terminal deoxynucleotidyl transferase (New England Biolabs) and deoxyadenosine triphosphate /deoxycytidine triphosphate according to the instruction of the manufacturer. The cDNA was then used as a template for a PCR using the AccuPrime Taq DNA polymerase (Invitrogen) and along with the primers RusV_323- as well as AAP and AP for the poly(C)- and poly(A)-elongated cDNA, respectively. Subsequently, a nested PCR using the primers AUAP and RusV_GSP1_M13 was done (for primer sequences, see Supplementary Table S2). For both PCRs, the recommended cycler protocol with each forty cycles was used. The final PCR products were cleaned with AMPure XP magnetic beads (Beckman Coulter) and prepared for sequencing using BigDye Terminator v1.1—Cycle Sequencing Kit (Applied Biosystems, Darmstadt, Germany) using the M13_rev primer. Sequencing reaction products were cleaned using NucleoSEQ columns (Macherey-Nagel), and sequencing was done on a 3500 Series Genetic Analyzer (Applied Biosystems), following the manufacturer’s instructions.

### 2.9 Phylogenetic analysis

Annotated full-length RusV genome sequences were aligned with publicly available RusV sequences using MAFFT (Katoh and Standley 2013) (version 7.450), and a maximum-likelihood phylogenetic tree was calculated using IQ-TREE (Nguyen et al. 2015) (version 2.2.0) running in automated model selection with 100,000 ultra-fast bootstrap replicates (Minh, Nguyen, and von Haeseler 2013). Phylogenetic relationships based on the 715-nt-long partial E1-encoding region were deduced using the same method as described earlier. We tested the full-length RusV genome sequence alignment for evidence of recombination using RDP5 (Martin et al. 2021) (version Beta 5.34).

### 2.10 Isolation-by-distance analysis

Isolation-by-distance (IBD) analysis was performed based on the 715-nt alignment of the RusV E1-coding region, which was also used for phylogenetic analysis using R language (R Core Team 2022) (version 4.1.2). IBD patterns would be expected if transmission of RusV between host populations is limited and evolutionary patterns are mostly governed by processes at local scales (Saxenhofer et al. 2017). The pairwise geographic distance between the sampling locations was calculated with R package ‘geodist’, and pairwise genetic distances between sequences were calculated using the Jukes and Cantor (JC69) model and pairwise deletion as implemented in the R package ‘dist.ml’. The significance of the correlation between the geographic distances and genetic distances was determined by a Mantel test between the two matrices using the R package ‘ade4’ with 9,999 permutations.

### 2.11 Sliding-window analysis

The alignment of all available complete RusV genome sequences and the deduced aa sequences was used for a sliding-window analysis using R (R Core Team 2022) (version 4.1.2). The mean JC69 distance was calculated between all sequences in sliding windows (window = 200 nt; step width = 50 nt) using the functions ‘slidingWindow’ and ‘dist.ml’ (R packages ‘spider’ and ‘phangorn’, respectively). The mean genetic distances were calculated for all sequences, based on the lineages defined in the phylogenetic analysis. Furthermore, the mean GC content was calculated within each window of the alignment using the ‘GC.content’ function (R package ‘ape’). From the full-genome alignment, the aa sequences of nsPP and sPP were deduced and aligned using MUSCLE (Edgar 2004) (version 3.8.425). In order to estimate aa exchanges across the viral proteins, the mean Jones-Taylor-Thornton (JTT) distance within all sequences was then calculated in a sliding window (window = 50 aa; step width = 15 aa). Conserved domains within nsPP and sPP were identified based on aa sequences from MN552442.2 with CD-Search using the default options (Marchler-Bauer and Bryant 2004). The probabilistic JC69 and JTT substitution models were applied as they tend to reflect the evolutionary background better than, e.g., p-distances (Saxenhofer et al. 2017).

### 2.12 Individual RusV infection risk

To analyze the factors affecting the individual RusV infection risk, a generalized linear model (GLM) with binomial error distribution was generated. Fixed factors included the trapping site, the year and season of trapping, individual sex (male/female), mass (in gram), and species richness (defined as the number of different species per trapping and site). Backward model selection was done using the function ‘drop1’ (R package ‘stats’). For categorical factors with more than three levels, a Tukey *post-hoc* analysis was performed. The 95 per cent confidence intervals of the RNA detection rate were estimated using the ‘exactci’ function (R package ‘exactci’). All analyses were done using R language (R Core Team 2022).

### 2.13 Histopathology and detection of RusV-specific RNA by ISH

Brain samples of five yellow-necked field mice (KS21-1231, KS21-1234, KS21-1237, KS21-1239 and KS21-1244) that had tested positive for RusV by RT-qPCR were available for histopathological analysis and routinely fixed in 4 per cent neutral buffered formaldehyde, paraffin embedded, and cut into 4 µm sections. In order to identify a potential inflammatory or degenerative reaction, hematoxylin and eosin (H&E) staining was performed for light microscopic evaluation. Consecutive brain sections were prepared for RusV-specific RNA ISH using RNAScope 2–5 HD Reagent Kit-Red (Advanced Cell Diagnostics, Hayward, CA, USA) according to the manufacturer’s instructions and as previously described (Bennett et al. 2020b; Pfaff et al. 2022). RusV RNA was detected with a custom-designed probe targeting the RusV nsPP open reading frame. On consecutive sections, a positive control probe against peptidylprolyl isomerase B (cyclophilin B, *PPIB*) gene and a negative control probe against dihydrodipicolinate reductase gene (*DapB*) were included for technical control. Brain tissue from an archived, non-infected C57BL/6 mouse served as a negative control. To detect subtle inflammation, we used immunohistochemistry and applied cluster of differentiation 3 (CD3) as a T-cell marker and ionized calcium-binding adaptor molecule 1 (IBA-1) as a marker for microglia and macrophages as previously described (Bennett et al. 2020b). In addition, archived material from two female yellow-necked field mice (KS19-0928 and KS20-1340) whose brain samples have already been evaluated (Bennett et al. 2020b) were included for H&E staining as well as ISH. The brain tissue served as a positive control, and the heart, lung, liver, spleen, small intestine, large intestine, adrenal gland (*n* = 1), and uterus with embryo were tested to verify whether RusV is found outside the central nervous system and is associated with changes in the tissues.

## 3. Results

### 3.1 Detection of RusV in yellow-necked field mice from multiple sites in northeastern Germany

In order to assess the geographic distribution of RusV in Germany and to identify potential reservoir hosts, a total of 1,264 small mammals of sixteen different species were collected at nineteen trapping sites spread over Germany (Fig. 1A and Table 1).

**Table 1.** Detection of Rustrela virus (RusV) RNA in small mammals from sites in Germany.

	North-east		Other	
	MV	BB	NI, HE, TH, and BY	Subtotal
Rodentia: Muridae				
Yellow-necked field mouse (*Apodemus flavicollis*)	**45/250 (18%)^a^**	**5/43 (11.6%)**	0/103	**50/396 (12.6%)**
Striped field mouse (*Apodemus agrarius*)	0/73^a^	0/21	–	0/94
Wood mouse (*Apodemus sylvaticus*)	0/31	0/5	0/19	0/55
Eurasian harvest mouse (*Micromys minutus*)	0/21	0/1	–	0/22
House mouse (*Mus musculus*)	0/57^a^	–	–	0/57
Norway rat (*Rattus norvegicus*)	0/16^a^	–	–	0/16
Rodentia: Cricetidae				
Field vole *(Microtus agrestis)*	0/130	–	–	0/130
Common vole (*Microtus arvalis*)	0/245	0/4	0/4	0/253
Bank vole (*Myodes glareolus*)	0/202	0/4	0/1	0/207
Eulipotyphla: Soricidae				
Bicolored white-toothed shrew (*Crocidura leucodon*)	–	–	0/1	0/1
Lesser white-toothed shrew (*Crocidura suaveolens*)	–	–	0/1	0/1
Eurasian water shrew *(Neomys fodiens)*	0/2	–	–	0/2
Common shrew *(Sorex araneus)*	0/60	–	0/13	0/73
Eurasian pygmy shrew *(Sorex minutus)*	0/3	–	0/2	0/5
Eulipotyphla: Erinaceidae				
European hedgehog (*Erinaceus europaeus*)	–	–	0/1	0/1
Eulipotyphla: Talpidae				
European mole *(Talpa europaea)*	–	–	0/1	0/1

MV, Mecklenburg-Western Pomerania; BB, Brandenburg; NI, Lower Saxony; HE, Hesse; TH, Thuringia; BY, Bavaria.

a Contains animals that were already described by Bennett et al. (2020a,b) and Pfaff et al. 2022.

RusV RNA detection is highlighted in bold.

The continuous rodent pest management in the initially affected zoo in northeastern Germany during 2020 and 2021 resulted in the collection of twenty-four yellow-necked field mice, two bank voles (*Myodes glareolus* syn. *Clethrionomys glareolus*), three Norway rats (*Rattus norvegicus*), and two common shrews (*Sorex araneus*). RT-qPCR analysis using the previously published RusV-specific RT-qPCR ‘Assay 1’ resulted in the detection of RusV RNA in seven of twenty-four yellow-necked field mice, whereas none of the other small mammals tested positive for RusV (Table 1; see later).

RusV screenings of small mammals from other locations were initially also performed using the ‘Assay 1’ RT-qPCR and revealed several RusV-positive yellow-necked field mice (data not shown). However, RusV sequences obtained from these RusV-positive animals showed several nt mismatches at the primer- and probe-binding sites, demonstrating the necessity for adaptation of the RT-qPCR. Therefore, a novel ‘Assay 1a’ was designed implementing modifications of the forward primer and probe of ‘Assay 1’ (Supplementary Table S2) to cover a broader range of sequences. For a comparative evaluation, twenty-eight RusV-positive RNA samples from different trapping sites and reflecting the genetic variability of RusV were tested in parallel with both assays (Supplementary Fig. S1). For samples originating from the originally affected zoo (corresponding to Lineage 1A; see later), comparable or slightly lower Cq values were achieved by ‘Assay 1a’ as compared to ‘Assay 1’. For the majority of samples from other trapping sites, clearly improved results were observed when using ‘Assay 1a’; one sample had even remained undetectable by ‘Assay 1’. However, for three samples, the Cq values of ‘Assay 1’ were lower by 1.1–3.7.

**Figure 2.**
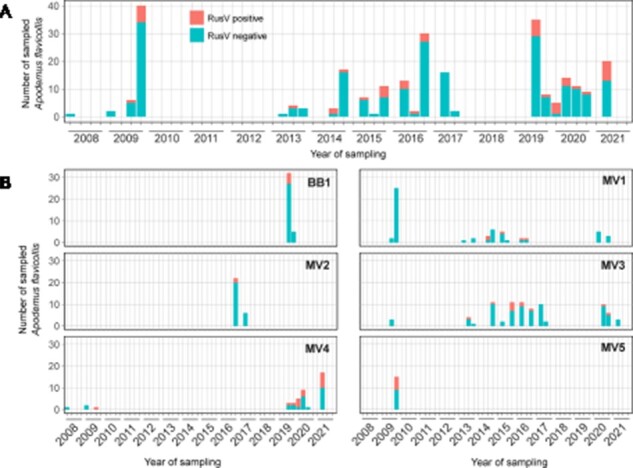
The temporal distribution of RusV detection, as tested by RusV-specific RT-qPCR, in yellow-necked field mouse (*Apodemus flavicollis*) populations. (A) The cumulative detection of RusV RNA at the study sites MV1–MV5 and BB1. The sampling time of RusV-positive and RusV-negative samples is shown as yearly quarters. (B) The trapping site–based detection of RusV RNA in yellow-necked field mice over the time of the study for locations BB1 and MV1–MV5.

Hence, the screening study was performed using the optimized RT-qPCR ‘Assay 1a’. RusV RNA was exclusively detected in yellow-necked field mice trapped in northeastern Germany (Fig. 1A and Table 1). Overall, 50 out of 293 (17.1 per cent) yellow-necked field mice sampled at six locations in northeastern Germany tested positive for RusV RNA (Fig. 1B). The detection rate at these locations varied between 8.8 per cent and 41.9 per cent. No other sympatric small mammal species were found RusV positive at any location including those sites where RusV-positive yellow-necked field mice were found (Fig. 1C). In general, RusV-positive individuals were sampled in northeastern Germany over a broad spectrum of timepoints, with the earliest detection in 2009 (Fig. 2A). This tendency is reflected also by results for trapping sites MV1 and MV3 (Fig. 2B).

In contrast, 146 small mammals from other regions of Germany, including 103 yellow-necked field mice and 19 wood mice, tested negative in the RT-qPCR ‘Assay 1a’ (Table 1). To confirm these results and exclude the possibility of genetically divergent RusV variants that are not detectable by ‘Assay 1a’, pools of brain-derived RNA from seventeen to twenty *Apodemus* mice per location from a limited number of sites were investigated by a metagenomic approach with a total of 14.2–22.5 million reads per pool for the original libraries (compare Fig. 1A and Supplementary Table S3). None of these reads—of either the original or the target-enriched sequencing library—showed any similarity with rubiviral sequences using diamond BLASTx search.

### 3.2 Ecological factors associated with RusV infections of yellow-necked field mice

The individual RusV infection risk for yellow-necked field mice was evaluated using binomial GLM analysis. During model selection, temporal pattern (year and season), sex, and site-specific species richness were eliminated from the global model. The final model included two significant factors (Supplementary Table S4). Body weight showed a positive association with infection risk, indicating that heavier individuals were more frequently infected (Supplementary Fig. S2A). In addition, *post-hoc* analysis revealed that individuals from the trapping site MV5 exhibited a significantly higher infection risk compared to trapping sites MV1, MV3, and BB1 (Supplementary Fig. S2B).

### 3.3 Genetically divergent RusV virus strains in northeastern Germany

**Figure 3.**
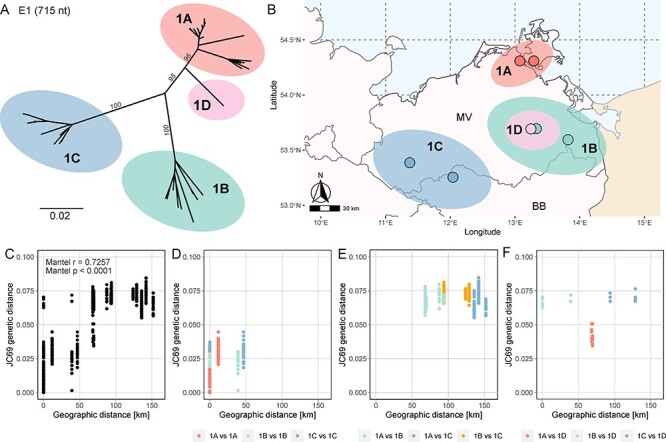
Phylogeographic clusters of RusV sequences in northeast Germany. (A) A 715-nt fragment of the gene coding for the E1 glycoprotein (sPP) was sequenced from forty RusV-positive yellow-necked field mice from six trapping sites. A nt sequence alignment of these sequences along with fourteen already published sequences of the same genomic region was used for maximum-likelihood phylogenetic reconstruction using IQ-TREE (version 2.2.0; model TN+F+G4; 100,000 ultra-fast bootstraps). The resulting phylogenetic tree can be divided into the phylogenetic lineages 1A, 1B, 1C, and 1D. Bootstrap values are indicated in italics and are only shown for major branches. (B) The map shows the sampling sites (dots) of RusV included in the phylogenetic analysis of the E1-coding fragment. The colors of the dots and areas around them correspond to the RusV phylogenetic lineage that was identified at the respective site. (C–F) Pairwise genetic distances (JC69) plotted against geographic distance between the respective sampling sites. (C) Subsets of all available pairwise comparisons were selected according to their phylogenetic lineage and intra- (D) and inter-lineage (E and F) relations are presented.

In total, thirty-seven and three partial E1-coding sequences with a length of 715 or 438 nt, respectively, were obtained. These sequences were combined with fourteen published RusV E1-coding sequences originating from the area where RusV was initially discovered (Bennett et al. 2020b; Pfaff et al. 2022). The phylogenetic analysis of the partial E1-coding sequences showed three well-supported phylogenetic clusters (bootstrap support values 95–100) designated as lineages 1A–1C (Fig. 3A). The sequence of a single sample was genetically divergent from lineages 1A–1C and was therefore designated as a potential fourth RusV lineage 1D (Fig. 3A).

The RusV lineages showed a strong association with geography (Fig. 3B, C; Mantel test *P* < 0.0001). The single sequence representing lineage 1D originated from a trapping site, where all other RusV-positive samples (*n* = 14), represented lineage 1B. Sequences from the same lineage (sequence identity: >95 per cent) were observed within less than 50 km from each other (Fig. 3D). At geographic distances of more than 50 km, different genetic lineages were observed. They showed >90  but <95 per cent nt sequence identity between them (Fig. 3E). Only the single available sequence of lineage 1D differed from this scheme in that it was found sympatrically to lineage 1B strains but was genetically more closely related to lineage 1A than to 1B and 1C (Fig. 3F).

**Figure 4.**
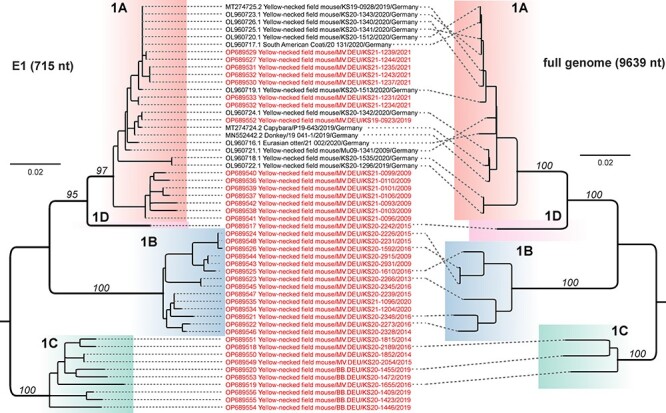
Congruence between phylogenetic reconstructions of RusV partial E1 and full-genome sequences. Phylogenetic trees were constructed based on alignments of fifty-four partial E1-coding (715 nt; left tree) and twenty-four complete RusV genome (9,639 nt; right tree) sequences. The partial E1-coding tree resembles that from Fig. 3A. For the full-genome alignment, a phylogenetic tree was calculated using maximum-likelihood reconstruction with IQ-TREE (version 2.2.0; model TN+F+I; 100,000 ultra-fast bootstraps). RusV sequences obtained in this study are depicted in red letters. The genetic lineages 1A, 1B, 1C, and 1D are highlighted. Bootstrap values are indicated in italics and are only shown for major branches. The dashed lines indicate the respective taxon for each branch tip.

Whole-genome sequencing was done for ten RusV-positive samples that represented lineages 1B (*n* = 6), 1C (*n* = 3), and 1D (*n* = 1), based on E1-coding sequences. Along with the already published RusV sequences from lineage 1A (*n* = 14), the full-genome alignment comprised twenty-four RusV complete genome sequences. The phylogenetic tree based on complete RusV genomes was congruent with the tree based on the partial E1-coding region and confirmed the presence of the genetically distinct phylogenetic lineages 1A–1D (Fig. 4). The pairwise nt sequence comparison revealed a nt sequence identity of 96.2–100 per cent within lineages and 92.0–95.3 per cent between lineages (Supplementary Fig. S3). We did not detect evidence of recombination between the different RusV lineages.

### 3.4 RusV genomes revealed highly divergent regions

**Figure 5.**
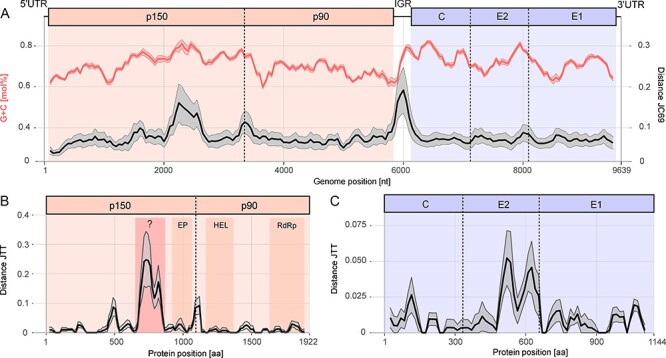
The genetic diversity within different regions of the RusV genome. (A) The RusV genome architecture is depicted, highlighting the coding region of the nsPP (left panel) with its mature peptides p150 and p90 as well as the coding region of the sPP (right panel) with its mature peptides capsid (C), glycoproteins E2 and E1. The coding region for nsPP and sPP are separated by an intergenic region (IGR). All available full-genome RusV sequences were aligned and analyzed using a sliding-window approach (window = 200 nt and step = 50 nt). The average GC content and genetic distance (JC69) along the alignment are depicted in red and black, respectively. The gray and light red ribbons show the standard deviation of GC content and genetic distance, respectively. (B and C) The genetic distances of the aa sequences of RusV nsPP (B) and sPP (C) were compared using a sliding-window approach (window = 50 aa and step = 15 aa) and the JTT model. The mean genetic distance between all sequences is highlighted in black and the standard deviation as gray ribbon. Regions of the polyproteins that contain conserved domains were deduced using CD-Search: endopeptidase (NP), RNA helicase (HEL), and RNA-directed RNA polymerase (RdRp). A region of especially high aa sequence diversity but unknown function was highlighted in red. In the RuV genome, this region contains the HVR and X macro domain.

A sliding-window analysis showed the reported extraordinary high GC content throughout the genome of RusV (Fig. 5A, red curve). The mean nt sequence diversity varied markedly along the RusV genome with regions of particularly high variability located in the p150 nsPP-coding region (position ∼2,100 to ∼2,600 nt) and in the intergenic region (Fig. 5A, black curve).

The overall mean aa sequence identity of nsPP (protease, helicase, and RNA-directed RNA polymerase) and sPP (capsid, E2, and E1 domains) was comparably high, averaging at 97.4 per cent and 98.9 per cent, respectively. Notably, a region of low aa sequence similarity was observed in nsPP, located between aa 694 and 841, which corresponds to the region with low nt sequence identity (Fig. 5B). No domain was identified within this specific region using CD-Search (Marchler-Bauer and Bryant 2004). For sPP, the highest aa sequence diversity was observed in the carboxy-terminal region of glycoprotein E2 (Fig. 5C).

### 3.5 RusV RNA detection in tissues of yellow-necked field mice

For each trapping location with RusV RNA detection, at least one positive yellow-necked field mouse was screened for viral RNA distribution in all available organs using the optimized RusV RT-qPCR ‘Assay 1a’. Consistently, the highest viral RNA load was observed within the central nervous system and/or spinal cord for all twenty-one analyzed yellow-necked field mice (Supplementary Fig. S4). For all animals with available brain samples, it showed the lowest Cq value (range: 19.4–29.2) of all tissue samples. In addition to the central nervous system, viral RNA was also detected in tissues of the peripheral nervous system, such as eyes, adrenal glands, and peripheral nerves. We sporadically detected very low RusV RNA loads in other tissues (Supplementary Fig. S4).

### 3.6 Histopathological evaluation of RusV infection in yellow-necked field mice

**Figure 6.**
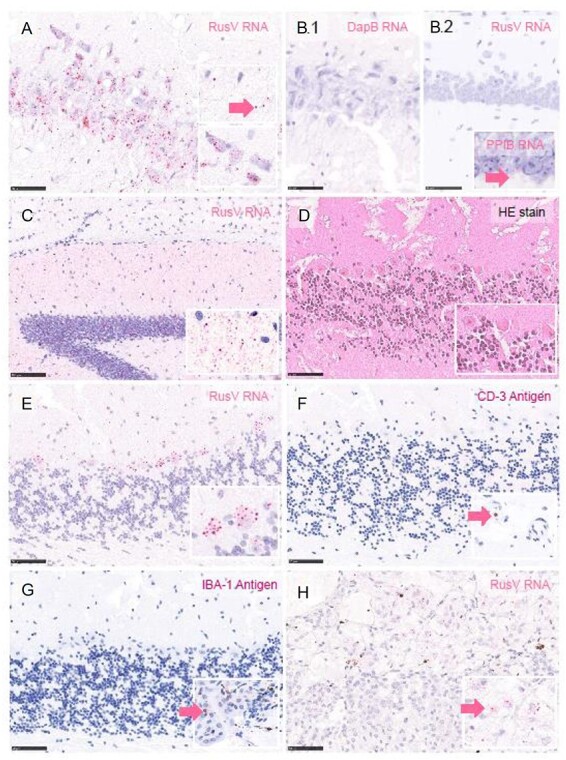
RNA ISH for RusV RNA detection, histopathology, and immune reaction in the brain and adrenal gland of yellow-necked field mice. RNA ISH using fast red chromogen and Mayer’s hematoxylin counterstain (A–C, E, and H), immunohistochemistry (IHC) using 3-amino-9-ethylcarbazole chromogen and Mayer’s hematoxylin counterstain (F and G), and H&E staining (D). ISH in hippocampus area with the detection of RusV RNA using probes against the nsPP-coding region showing chromogenic labeling mainly in neuronal cell bodies (lower inset) and scattered within the neuropil (upper inset with arrow), mouse KS21-1244 (A). ISH showing no labeling with a negative control probe against bacterial dihydrodipicolinate reductase (DapB) on the brain of mouse KS21-1244 (B1) and no chromogenic reaction after hybridization with RusV nsPP probe on a negative control section from archived mouse brain but scattered positive reactions with the positive control probe peptidylprolyl isomerase B (PPIB, inset with arrow) (B2). ISH with widespread RusV RNA labeling in the neuropil of the hippocampus area of mouse KS20-1341 (C with inset). H&E staining of the cerebellum with lack of obvious inflammatory reaction or degeneration of neurons (D with inset) in areas with intraneuronal RusV RNA detection using ISH in mouse KS19-0928 (E with inset). On consecutive brain sections, IHC failed to detect CD3-positive T cells (F, arrow displays intravascular CD3 labeling in a different area) or IBA-1-labeled microglia/macrophages (G, arrow displays IBA-1 labeling in a different area). ISH with multifocal RusV RNA detection in medullary chromaffin cells of the adrenal gland, mouse KS19-0928 (H inset with arrow). Scale bars 50 µm (A, B, and D–H) or 100 µm (C).

Brain samples were available for histopathology and detection of RusV RNA by ISH for seven RusV-positive mice, including five new and two previously published individuals (Bennett et al. 2020b). In general, the tissues showed moderate-to-severe autolytic changes and freezing artifacts. RNA ISH confirmed RusV nsPP-encoding RNA mainly in neuronal cell bodies but also within the neuropil of the cerebrum and cerebellum (Fig. 6A–C, with technical controls). Due to the chromogenic labeling dispersed in the neuropil (Fig. 6C), we cannot exclude that also glial cells (e.g. microglia and astrocytes) were affected. Histopathology did not reveal any evidence of inflammation or degeneration (Fig. 6D) in areas with RusV RNA detection (Fig. 6E). To be able to detect also subtle inflammation, we used IHC and applied markers for T cells (CD-3) and microglial cells/macrophages (IBA-1). IHC did not identify any T lymphocyte (Fig. 6F) or microglial cell/macrophage (Fig. 6G) aggregation, neither perivascularly nor (sub-)meningeally nor within the neuropil. However, it must be noted that the overall chromogenic labeling quality and the number of labeled cells might have been affected by autolysis and/or freezing, showing only overall very few immuno-positive cells (Fig. 6, insets in G and H). In the brain, single T-cell detection, intravascularly and also within the neuropil, was within normal limits. IBA-1 labels not only infiltrating macrophages and activated microglia but also non-activated, resident microglia. Thus, a widespread IBA-1 immunoreactivity was expected but not found in the brains tested.

Furthermore, RNA ISH was performed on a broader range of tissue samples from two mice (KS19-0928 and KS20-1340; Bennett et al. 2020b). ISH detected RusV RNA in the medullary chromaffin cells of the adrenal gland (Fig. 6H) in one mouse. All remaining tissues including the heart, lung, liver, spleen, small intestine, large intestine, and uterus with embryos tested negative. Based on H&E staining, a mild, chronic, non-suppurative, interstitial nephritis was found in both mice. However, the etiology and pathogenesis of this finding remain unexplained, and this is likely to be a background finding. The detection of an acute, diffuse, alveolar edema in the lungs of both animals was interpreted to be an agonal finding and thus non-related to RusV infection.

## 4. Discussion

### 4.1 Yellow-necked field mice as a reservoir for RusV

This study indicates the continuous presence of genetically diverse RusV lineages in Germany in its presumed reservoir: the yellow-necked field mouse. The RT-qPCR testing and target enrichment-HTS-based analysis of yellow-necked field mice and related wood mice suggest a heterogeneous distribution of RusV and perhaps the absence of RusV in the analyzed hosts from other regions in Germany. However, recent studies on domestic cats and captive lions confirmed the presence of RusV in other parts of Germany, such as Lower Saxony, North Rhine-Westphalia, and Saxony (de Le Roi et al. 2023; Matiasek et al. 2023).

Molecular investigations of various rodent species at multiple sites with an exclusive detection of RusV RNA in yellow-necked field mice and absence in sympatric other species indicate the role as the most likely reservoir of RusV. We found the RusV RNA detection rate to be positively correlated with body weight. Since weight is usually positively correlated with age in *Apodemus* species (Adamczewska-Andrzejewska 1973; Balčiauskienė, Balčiauskas, and Mažeikytė 2004), this may indicate an increased probability of detection with age and might, thus, be indicative of a persistent infection. An age dependency of infection rate was also observed for other rodent-borne pathogens, such as vole-associated orthohantaviruses and *Leptospira* spp. (Jeske et al. 2021; Schmidt et al. 2021). The persistence of infection represents additional support for the presumed reservoir function of yellow-necked field mice. In addition, histopathological analyses of brain tissue of RusV-infected yellow-necked field mice failed to detect any signs of inflammation (see later), a phenomenon that also may hint to the presumed reservoir function. Of note, the likely absence of disease in RusV-infected yellow-necked field mice is in contrast to the findings of disease in spillover hosts, including a rodent species (capybara). Based on our dataset, we could not exclude that a spillover infection of rodents other than the yellow-necked field mouse may also result in the disease, but in wildlife situations, this may not be seen due to rapid action of predators.

In our study, the improved RT-qPCR ‘Assay 1a’ proved to have a higher sensitivity for RusV RNA detection compared to the initial ‘Assay 1’ (Bennett et al. 2020b). The lack of RusV RNA detection by this optimized assay and a metagenomic approach with target enrichment may indicate the absence of RusV or RusV-like viruses in the examined *Apodemus* species outside northeastern Germany. However, the investigated brain pools represented only five trapping sites and comprise about twenty animals each. Hence, a broader and more systematic sampling may be required to analyze the distribution of RusV in Germany and neighboring countries. As demonstrated previously, the used workflow based on target enrichment HTS largely improved the chance to determine almost complete RusV genomes (Pfaff et al. 2022).

### 4.2 Evolution of RusV within its reservoir populations

The presence of geographically separated genetic lineages of RusV from yellow-necked field mice might indicate the stable and continuous presence of RusV that allowed independent evolutionary processes in isolated host populations. This is also supported by the fact that sequences of the same genetic lineages can be found over long periods of time at the same geographic location. In contrast, RusV lineage 1D, sampled from a single yellow-necked field mouse, represented an exception as it was found in a location where all other RusV-positive samples represented lineage 1B. This could indicate that the geographic range of RusV lineages is more widespread and may even be sympatric. It might be possible that 1D descended from a common ancestor with lineage 1A and was then geographically separated by migration of some infected individuals or the transportation of mice by predators. This could explain the comparably low genetic distance between lineages 1A and 1D despite the relatively large geographic distance.

Analogous to the correlation of genetic and geographic distance for RusV lineages using IBD (Fig. 3), a similar correlation has been observed for the genetic relations between yellow-necked field mouse populations in northeastern Poland (Czarnomska et al. 2018). It has also been shown that landscape patterns including natural borders such as lakes and rivers can have a profound impact on the genetic patterns within populations of the yellow-necked field mouse (Gortat et al. 2010). As a forest specialist, the ability to disperse is greatly influenced by the connectivity of the surrounding landscape, and a mosaic of fragmented forest patches could further limit gene flow between local populations (Gortat et al. 2010). The home range size of yellow-necked field mice is between ∼1,500 and ∼2,200 m^2^ with generally larger ranges for male animals (Montgomery 1977). The home ranges of individuals overlap, but centers of activity are typically separated (Montgomery 1977). Individuals may move between patches of woodland that are separated by 100 m, and during winter, individuals may disperse up to 300 m (Rajska-Jurgiel 1992). The prominent geographic clustering of the different RusV lineages supports the evolution of the virus in local yellow-necked field mouse populations with local transmission cycles and restricted spreading into other populations. Future studies have to prove a co-separation of RusV and its host by more detailed phylogeographic analyses of the yellow-necked field mouse reservoir (see e.g Saxenhofer et al. 2019; Saxenhofer et al. 2022), based on nuclear and mitochondrial gene analysis as previously reported (Michaux et al. 2004).

### 4.3 The sequence variability in the RusV genome is heterogeneously distributed

By analyzing the genetic diversity along the RusV genome and the encoded polyproteins, we observed two regions of particularly high variability: the non-coding intergenic region and a stretch within the p150 nsPP-coding region. The latter corresponds to a higher aa sequence variability within a single region toward the C-terminus of p150, whose function remains speculative. Interestingly, part of the higher nt sequence variability corresponds to regions of higher GC content. The genomic architecture and coding potential of RusV are assumed to be similar to that of RuV (Bennett et al. 2020b; Pfaff et al. 2022). The p150 mature peptide of RuV contains three regions toward the C-terminus: the so-called ‘hypervariable’ region (HVR) (Hofmann et al. 2003), the X domain, and the protease. While the coding nt sequences of the X domain and protease are well conserved within the RuV genotypes, the HVR is highly diverse and has a high substitution rate (Zhou, Ushijima, and Frey 2007; Abernathy et al. 2013). Also, the codon usage, GC content, and processes of natural selection of the HVR seem to be distinct from other genome regions of RuV (Zheng et al. 2003; Zhou et al. 2012). In detail, the plasticity of the RuV HVR may evolve under natural selection that favors structural stability of the genome and high GC content (Zhou et al. 2012).

We identified a genetically similar region in RusV that shares features with the RuV HVR, such as the location within the p150 coding region, a maximum in GC content (Zhou et al. 2012), and the low level of conservation across all analyzed RusV samples (Zhou, Ushijima, and Frey 2007). However, similar to RuV, the function of the potential HVR and the reason for its high substitution rate are currently unknown. It can be speculated that a comparable HVR may also be found in other related matonavirus genomes and therefore novel viruses should be screened for the presence of an HVR in order to identify common genetic patterns that may clarify its biological function (Shi et al. 2018; Geoghegan et al. 2021; Grimwood, Holmes, and Geoghegan 2021).

### 4.4 RusV detection in yellow-necked field mice is not associated with meningoencephalitis

In line with previous analyses (Bennett et al. 2020b), histopathological evaluation of brain samples of RusV-positive yellow-necked field mice did not indicate inflammation despite the detection of the viral genome in neurons by RNA ISH. However, the implications of this finding are limited by the low number of animals tested (*n* = 5 new cases in this study, *n* = 6 in Bennett et al. 2020b), autolysis, and freezing artifacts. In contrast to the initially reported cases of RusV-associated meningoencephalitis with neurologic disease in zoo animals (Bennett et al. 2020b; Pfaff et al. 2022), all mice were collected during rodent monitoring. Thus, the development of a meningoencephalitis at a later time point cannot be excluded.

In accordance with RT-qPCR data, ISH confirmed RusV RNA in the brains of mice. The detection of RusV RNA in medullary cells of the adrenal gland in one of the two analyzed animals is of particular interest. These cells represent a modified sympathetic ganglion of the autonomic nervous system (Carmichael 1997), which is innervated by preganglionic sympathetic neurons having their cell bodies in the spinal cord. Ultimately, we cannot exclude hematogenous dissemination but consider a neural route more likely. Whether the infection of the adrenal gland is a consequence of the infection of the central nervous system or vice versa remains speculative. However, both routes would require dissemination via the peripheral nervous system, which should be evaluated in upcoming studies in more detail. In contrast to the RT-qPCR results, ISH did not detect RusV in any other tissue tested, which may indicate a lower sensitivity of RNA detection by ISH.

## 5. Conclusions

The detection of encephalitis cases in mammals of various species, including zoo animals, and the persistent occurrence of RusV within local populations of yellow-necked field mice indicates a continuous threat of RusV infection. The here described geographical distribution of RusV needs to be re-evaluated by further extensive studies that will benefit from the presented workflow. These further studies should particularly include regions where RusV was recently detected in spillover hosts such as zoo animals and domestic cats. Pest rodent investigations and passive surveillance of zoo animals in zoological gardens should be continued for a risk assessment and improvement of pest management. Future serological studies should also evaluate a zoonotic potential of RusV in putative risk groups that have been in contact with yellow-necked field mice, such as employees of zoos and forest workers but also by RusV screening of encephalitis cases of unknown origin. Finally, ecological studies on yellow-necked field mouse populations should test for the potential influences of population dynamics on the frequency of RusV infections within the reservoir and thereby the probability of transmission to other mammals. The multiple detection of RusV RNA in several reservoir populations may facilitate future virus isolation in cell culture or animal experiments.

## Supplementary Material

vead048_Supp

## Data Availability

All novel RusV sequences were uploaded to GenBank and are available under the accessions OP689517–OP689556 (PRJNA790443).

## References

[R1] Abernathy E. et al. (2013) ‘Analysis of Whole Genome Sequences of 16 Strains of Rubella Virus from the United States, 1961–2009’, *Virology Journal*, 10: 32. 10.1186/1743-422X-10-32 PMC3574052 23351667

[R2] Adamczewska-Andrzejewska K. (1973) ‘Growth, Variations and Age Criteria in *Apodemus agrarius* (Pallas, 1771)’, *Acta Theriologica*, 18: 353–94.

[R3] Balčiauskienė L. , Balčiauskas L., and Mažeikytė J. R. (2004) ‘Sex- and Age Related Differences in Tooth Row Length of Small Mammals: Mice’, *Acta Zoologica Lituanica*, 14: 54–65.

[R4] Bankevich A. et al. (2012) ‘SPAdes: A New Genome Assembly Algorithm and Its Applications to Single-Cell Sequencing’, *Journal of Computational Biology*, 19: 455–77. 22506599 10.1089/cmb.2012.0021 PMC3342519

[R5] Bennett A. J. et al. (2020a) ‘Author Correction: Relatives of Rubella Virus in Diverse Mammals’, *Nature*, 588: E2. 10.1038/s41586-020-2897-1 33199919

[R36] ——— et al. (2020b) ‘Relatives of Rubella Virus in Diverse Mammals’, *Nature*, 586: 424–8. 33029010 10.1038/s41586-020-2812-9 PMC7572621

[R6] Buchfink B. , Xie C., and Huson D. H. (2015) ‘Fast and Sensitive Protein Alignment Using DIAMOND’, *Nature Methods*, 12: 59–60. 25402007 10.1038/nmeth.3176

[R7] Carmichael S. W. (1997) ‘The Adrenal Medulla’, in E. Edward Bittar (ed) *Molecular and Cellular Endocrinology*, Vol. 10, pp. 1–684. Elsevier.

[R8] Chen R. et al. (2018) Create a New Family *Matonaviridae* to Include the Genus *Rubivirus*, Removed from the Family *Togaviridae* <https://talk.ictvonline.org/ictv/proposals/2018.013S.A.v3.Matonaviridae.zip> accessed 28 July 2021.

[R9] Czarnomska S. D. et al. (2018) ‘Regional and Local Patterns of Genetic Variation and Structure in Yellow-Necked Mice – The Roles of Geographic Distance, Population Abundance, and Winter Severity’, *Ecology and Evolution*, 8: 8171–86. 30250693 10.1002/ece3.4291 PMC6145024

[R10] Das P. K. , Kielian M., and Parrish C. R. (2021) ‘The Enigmatic Capsid Protein of an Encephalitic Rubivirus’, *Journal of Virology*, 95: 10–1128. 10.1128/JVI.02294-20 PMC8103679 33472932

[R11] de Le Roi M. et al. (2023) ‘Rustrela Virus as Putative Cause of Nonsuppurative Meningoencephalitis in Lions’, *Emerging Infectious Diseases*, 29: 1042–5. 37081716 10.3201/eid2905.230172 PMC10124629

[R12] Drewes S. et al. (2017) ‘Host-Associated Absence of Human Puumala Virus Infections in Northern and Eastern Germany’, *Emerging Infectious Diseases*, 23: 83–6. 27983499 10.3201/eid2301.160224 PMC5176216

[R13] Edgar R. C. (2004) ‘MUSCLE: A Multiple Sequence Alignment Method with Reduced Time and Space Complexity’, *BMC Bioinformatics*, 5: 113. 10.1186/1471-2105-5-113 PMC517706 15318951

[R14] Geoghegan J. L. et al. (2021) ‘Virome Composition in Marine Fish Revealed by Meta-Transcriptomics’, *Virus Evolution*, 7: veab005. 10.1093/ve/veab005 PMC7887440 33623709

[R15] Gortat T. et al. (2010) ‘Landscape Pattern and Genetic Structure of a Yellow-Necked Mouse *Apodemus flavicollis* Population in North-Eastern Poland’, *Acta Theriologica*, 55: 109–21.

[R16] Grimwood R. M. , Holmes E. C., and Geoghegan J. L. (2021) ‘A Novel Rubi-Like Virus in the Pacific Electric Ray (*Tetronarce californica*) Reveals the Complex Evolutionary History of the Matonaviridae’, *Viruses*, 13: 585. 10.3390/v13040585 PMC8067182 33807136

[R17] Hoffmann B. et al. (2006) ‘A Universal Heterologous Internal Control System for Duplex Real-Time RT-PCR Assays Used in a Detection System for Pestiviruses’, *Journal of Virological Methods*, 136: 200–9. 16806503 10.1016/j.jviromet.2006.05.020

[R18] Hofmann J. et al. (2003) ‘Phylogenetic Analysis of Rubella Virus Including New Genotype I Isolates’, *Virus Research*, 96: 123–8. 12951272 10.1016/s0168-1702(03)00180-1

[R19] Jeske K. et al. (2021) ‘Hantavirus–*Leptospira* Coinfections in Small Mammals from Central Germany’, *Epidemiology and Infection*, 149: e97. 10.1017/S0950268821000443 PMC8101269 33612134

[R20] Katoh K. , and Standley D. M. (2013) ‘MAFFT Multiple Sequence Alignment Software Version 7: Improvements in Performance and Usability’, *Molecular Biology and Evolution*, 30: 772–80. 23329690 10.1093/molbev/mst010 PMC3603318

[R21] Knipe, D. M., and Howley, P. M. eds (2007) *Fields Virology: Rubella Virus*, 5th edn. Lippincott Williams & Wilkins: Philadelphia.

[R22] Mankertz A. et al. (2022) ‘ICTV Virus Taxonomy Profile: *Matonaviridae* 2022’, *Journal of General Virology*, 103: 001817. 10.1099/jgv.0.001817 PMC12643105 36748520

[R23] Marchler-Bauer A. , and Bryant S. H. (2004) ‘CD-Search: Protein Domain Annotations on the Fly’, *Nucleic Acids Research*, 32: W327–31. 15215404 10.1093/nar/gkh454 PMC441592

[R24] Martin D. P. et al. (2021) ‘RDP5: A Computer Program for Analyzing Recombination In, and Removing Signals of Recombination From, Nucleotide Sequence Datasets’, *Virus Evolution*, 7: veaa087. 10.1093/ve/veaa087 PMC8062008 33936774

[R25] Matiasek K. et al. (2023) ‘Mystery of Fatal ‘Staggering Disease’ Unravelled: Novel Rustrela Virus Causes Severe Meningoencephalomyelitis in Domestic Cats’, *Nature Communications*, 14: 624. 10.1038/s41467-023-36204-w PMC9899117 36739288

[R26] Michaux J. R. et al. (2004) ‘Phylogeographic History of the Yellow-Necked Fieldmouse (*Apodemus flavicollis*) in Europe and in the near and Middle East’, *Molecular Phylogenetics and Evolution*, 32: 788–98. 15288056 10.1016/j.ympev.2004.02.018

[R27] Minh B. Q. , Nguyen M. A. T., and von Haeseler A. (2013) ‘Ultrafast Approximation for Phylogenetic Bootstrap’, *Molecular Biology and Evolution*, 30: 1188–95. 23418397 10.1093/molbev/mst024 PMC3670741

[R28] Montgomery W. I. (1977) ‘Studies on the Ecology of Two Sympatric Species of Apodemus (Rodentia: Muridae)’.

[R29] Nguyen L.-T. et al. (2015) ‘IQ-TREE: A Fast and Effective Stochastic Algorithm for Estimating Maximum-Likelihood Phylogenies’, *Molecular Biology and Evolution*, 32: 268–74. 25371430 10.1093/molbev/msu300 PMC4271533

[R30] Oker-Blom C. (1984) ‘The Gene Order for Rubella Virus Structural Proteins Is NH2-C-E2-E1-COOH’, *Journal of Virology*, 51: 354–8. 6748161 10.1128/jvi.51.2.354-358.1984 PMC254445

[R31] Oker-Blom C. et al. (1984) ‘Rubella Virus 40S Genome RNA Specifies a 24S Subgenomic mRNA that Codes for a Precursor to Structural Proteins’, *Journal of Virology*, 49: 403–8. 6694262 10.1128/jvi.49.2.403-408.1984 PMC255479

[R32] Oker-Blom C. , Jarvis D. L., and Summers M. D. (1990) ‘Translocation and Cleavage of Rubella Virus Envelope Glycoproteins: Identification and Role of the E2 Signal Sequence’, *Journal of General Virology*, 71: 3047–53. 2273395 10.1099/0022-1317-71-12-3047

[R33] Pfaff F. et al. (2022) ‘Revisiting Rustrela Virus: New Cases of Encephalitis and a Solution to the Capsid Enigma’, *Microbiology Spectrum*, 10: e0010322. 10.1128/spectrum.00103-22 PMC9045237 35384712

[R34] Rajska-Jurgiel E. (1992) ‘Demography of Woodland Rodents in Fragmented Habitat’, *Acta Theriologica*, 37: 73–90.

[R35] R Core Team . (2022) R: A Language and Environment for Statistical Computing, Vienna, Austria. <http://www.R-project.org/>.

[R38] Saxenhofer M. et al. (2022) ‘Host Genetic Factors Associated with the Range Limit of a European Hantavirus’, *Molecular Ecology*, 31: 252–65. 34614264 10.1111/mec.16211 PMC9298007

[R41] ——— et al. (2019) ‘Secondary Contact between Diverged Host Lineages Entails Ecological Speciation in a European Hantavirus’, *PLoS Biology*, 17: e3000142. 10.1371/journal.pbio.3000142 PMC6382107 30785873

[R37] ——— et al. (2017) ‘Revised Time Scales of RNA Virus Evolution Based on Spatial Information’, *Proceedings. Biological Sciences*, 284: 20170857. 10.1098/rspb.2017.0857 PMC5563803 28794221

[R39] Schlegel M. et al. (2012) ‘Molecular Identification of Small Mammal Species Using Novel Cytochrome B Gene-Derived Degenerated Primers’, *Biochemical Genetics*, 50: 440–7. 22193288 10.1007/s10528-011-9487-8

[R40] Schmidt E. et al. (2021) ‘Influence of Season, Population and Individual Characteristics on the Prevalence of *Leptospira* spp. In Bank Voles in North-West Germany’, *Biology (Basel)*, 10: 933. 10.3390/biology10090933 PMC8466531 34571810

[R42] Shi M. et al. (2018) ‘The Evolutionary History of Vertebrate RNA Viruses’, *Nature*, 556: 197–202. 29618816 10.1038/s41586-018-0012-7

[R43] Stubbe W. (2012) ‘Beitrag Zur Kleinsäugerfauna der Insel Rügen’, *Säugetierkundliche Informationen*, 44, 251–89.

[R44] Voss A. et al. (2022) ‘Rustrela Virus Infection – An Emerging Neuropathogen of Red-necked Wallabies (*Macropus rufogriseus*)’, *Transboundary and Emerging Diseases*, 69: 4016–21. 36135593 10.1111/tbed.14708

[R45] Weber de Melo V. et al. (2015) ‘Spatiotemporal Dynamics of Puumala Hantavirus Associated with Its Rodent Host, *Myodes glareolus*’, *Evolutionary Applications*, 8: 545–59. 26136821 10.1111/eva.12263 PMC4479511

[R46] Wylezich C. et al. (2018) ‘A Versatile Sample Processing Workflow for Metagenomic Pathogen Detection’, *Scientific Reports*, 8: 13108. 10.1038/s41598-018-31496-1 PMC6117295 30166611

[R47] Zheng D.-P. et al. (2003) ‘Global Distribution of Rubella Virus Genotypes’, *Emerging Infectious Diseases*, 9: 1523–30. 14720390 10.3201/eid0912.030242 PMC3034328

[R48] Zhou Y. et al. (2012) ‘Analysis of Base and Codon Usage by Rubella Virus’, *Archives of Virology*, 157: 889–99. 22322905 10.1007/s00705-012-1243-9

[R49] Zhou Y. , Ushijima H., and Frey T. K. (2007) ‘Genomic Analysis of Diverse Rubella Virus Genotypes’, *Journal of General Virology*, 88: 932–41. 17325367 10.1099/vir.0.82495-0

