# Supplementary material for: Continuous presence of genetically diverse rustrela virus lineages in yellow-necked field mouse reservoir populations in northeastern Germany
Source: Virus Evol. 2023 Jul 28;9(2):vead048. doi: 10.1093/ve/vead048 (PMC10516363; doi:10.1093/ve/vead048)
Supplement: vead048_Supp [file vead048_supp.zip › Nippert_Rustrela virus Supplement_endfinal_corrected_2023 (1).pdf]

Supplementary Data for:

Continuous presence of genetically diverse rustrela virus lineages in yellow-necked field mouse reservoir populations in northeastern Germany

Nippert *et al.* 2023

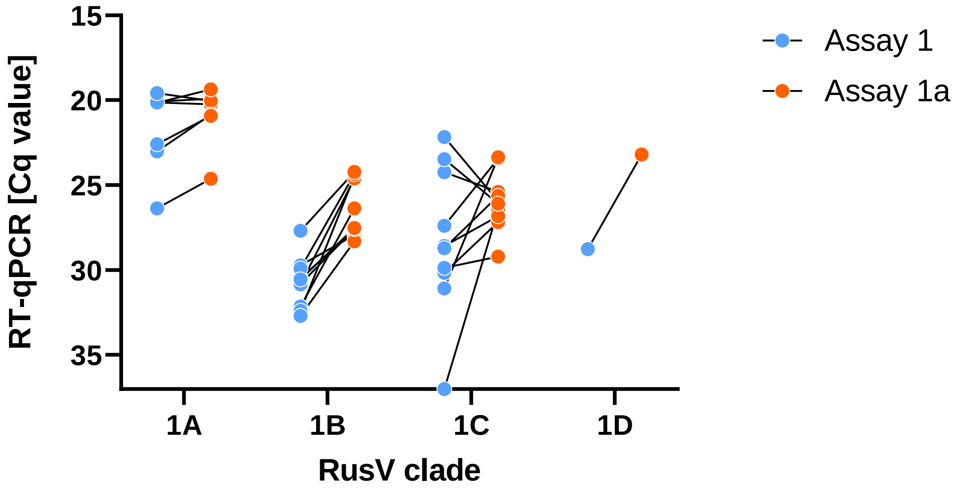

Supplementary Figure S1: Comparison of two RusV-specific RT-qPCR assays. Twenty-eight selected samples representing all four genetic lineages were tested in parallel with either the previously published "Assay 1" (Bennett et al., 2020a,b) or with the modified "Assay 1a" that was designed to detect a broader range of RusV sequences from northeastern Germany. Results are presented as cycle of quantification (Cq) values. The detection limit of the assay is at a Cq value of 37; the scaling of the y-axis starts accordingly.

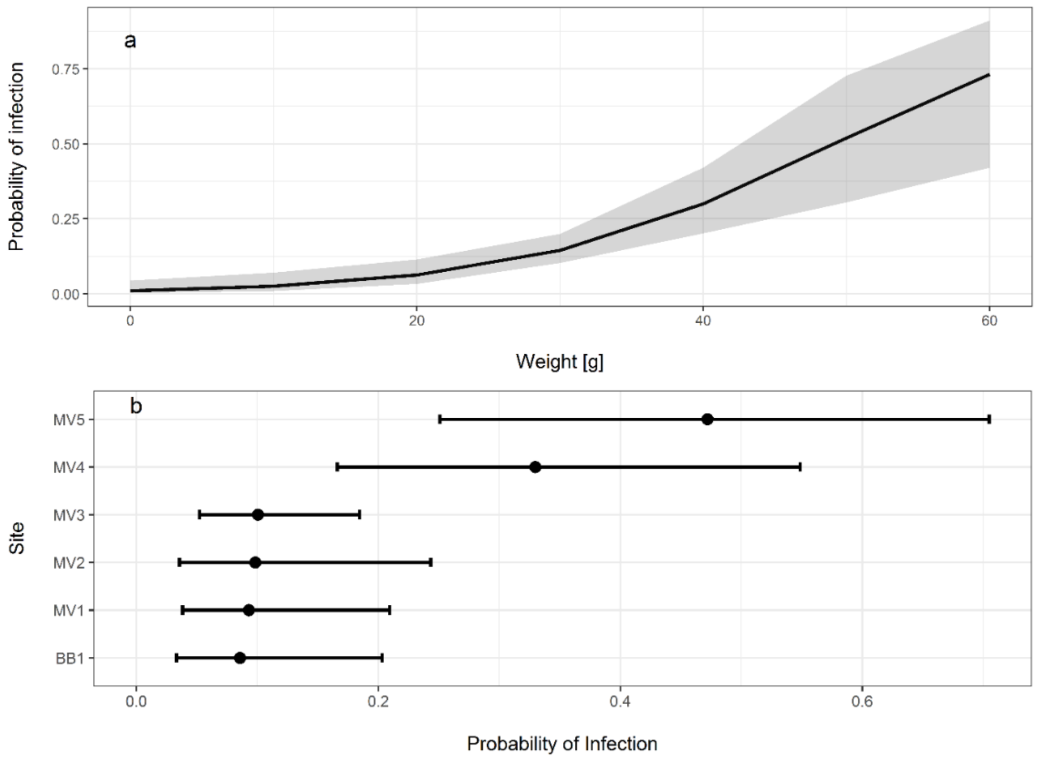

Supplementary Figure S2: Ecological factors of the RusV infection risk in yellow-necked field mice. In order to analyse ecological factors associated with RusV infections of yellow-necked field mice a generalized linear model (GLM) with binomial error distribution was generated. Fixed factors included the trapping site, the year and season of trapping, sex (male/female) and mass (in gram) of the individuals and species richness (defined as number of different species per trapping and site). (**a**) Individual body weight showed a positive association with infection risk. (**b**) *Post hoc* analysis revealed that individuals from the trapping site MV5 exhibited a significantly higher infection risk compared to trapping sites MV1, MV3 and BB1.

Lineage		1A														1B						1C			1D
	Strain	MN552442.2	MT274724.2	MT274725.2	OL960716.1	OL960717.1	OL960718.1	OL960719.1	OL960720.1	OL960721.1	OL960722.1	OL960723.1	OL960724.1	OL960725.1	OL960726.1	**KS20-1592**	**KS20-1610**	**KS20-2226**	**KS20-2266**	**KS20-2273**	**KS20-2346**	**KS20-1455**	**KS20-1655**	**KS20-2189**	**KS20-2242**
1A	MN552442.2 Donkey/19_041-1/2019/Germany																								
	MT274724.2 Capybara/P19-643/2019/Germany	99.8																							
	MT274725.2 Yellow-necked field mouse/KS19-928/2019/Germany	99.1	99.0																						
	OL960716.1 Eurasian otter/21_002/2020/Germany	99.0	98.9	98.8																					
	OL960717.1 South American Coati/20_131/2020/Germany	99.0	98.9	99.8	98.8																				
	OL960718.1 Yellow-necked field mouse/KS20-1535/2020/Germany	97.7	97.6	97.5	97.4	97.4																			
	OL960719.1 Yellow-necked field mouse/KS20-1513/2020/Germany	99.1	98.9	99.6	98.8	99.6	97.5																		
	OL960720.1 Yellow-necked field mouse/KS20-1512/2020/Germany	99.0	99.0	99.8	98.8	100.0	97.5	99.6																	
	OL960721.1 Yellow-necked field mouse/Mu09-1341/2009/Germany	99.3	99.2	99.1	99.0	99.0	97.7	99.1	99.0																
	OL960722.1 Yellow-necked field mouse/KS20-1296/2019/Germany	97.7	97.7	97.5	97.4	97.5	100.0	97.5	97.5	97.7															
	OL960723.1 Yellow-necked field mouse/KS20-1343/2020/Germany	99.1	99.0	99.8	98.8	99.9	97.5	99.6	99.9	99.1	97.5														
	OL960724.1 Yellow-necked field mouse/KS20-1342/2020/Germany	99.8	99.8	99.1	99.0	99.0	97.7	99.0	99.0	99.3	97.7	99.0													
	OL960725.1 Yellow-necked field mouse/KS20-1341/2020/Germany	99.0	98.9	99.8	98.8	99.9	97.5	99.6	99.9	99.0	97.5	99.9	99.0												
	OL960726.1 Yellow-necked field mouse/KS20-1340/2020/Germany	99.1	99.0	99.8	98.8	99.8	97.5	99.6	99.8	99.1	97.5	99.8	99.0	99.8											
1B	**RusVs/yellow-necked field mouse/MV.DEU/KS20-1592/2016**	92.4	92.3	92.4	92.3	92.4	92.5	92.3	92.5	92.3	92.5	92.5	92.3	92.5	92.4										
	**RusVs/yellow-necked field mouse/MV.DEU/KS20-1610/2016**	92.5	92.4	92.5	92.4	92.5	92.6	92.5	92.5	92.5	92.6	92.5	92.5	92.5	92.5	98.3									
	**RusVs/yellow-necked field mouse/MV.DEU/KS20-2226/2015**	92.4	92.3	92.4	92.3	92.4	92.5	92.4	92.5	92.3	92.5	92.5	92.3	92.5	92.4	99.9	98.3								
	**RusVs/yellow-necked field mouse/MV.DEU/KS20-2266/2013**	92.4	92.4	92.4	92.3	92.4	92.4	92.3	92.4	92.3	92.4	92.4	92.4	92.4	92.4	96.6	96.7	96.6							
	**RusVs/yellow-necked field mouse/MV.DEU/KS20-2273/2016**	92.3	92.3	92.3	92.2	92.3	92.3	92.3	92.3	92.2	92.3	92.3	92.2	92.3	92.3	96.5	96.6	96.4	97.5						
	**RusVs/yellow-necked field mouse/MV.DEU/KS20-2346/2016**	92.2	92.2	92.1	92.0	92.1	92.1	92.1	92.1	92.1	92.1	92.1	92.1	92.1	92.1	96.2	96.3	96.2	97.4	98.4					
1C	**RusVs/yellow-necked field mouse/BB.DEU/KS20-1455/2019**	92.9	92.8	93.0	92.9	92.9	92.7	92.9	92.9	92.8	92.7	93.0	92.8	93.0	93.0	92.3	92.6	92.3	92.4	92.2	92.3				
	**RusVs/yellow-necked field mouse/MV.DEU/KS20-1655/2016**	92.8	92.8	92.8	92.8	92.8	92.7	92.8	92.8	92.8	92.7	92.8	92.8	92.8	92.8	92.4	92.6	92.4	92.6	92.3	92.3	96.4			
	**RusVs/yellow-necked field mouse/MV.DEU/KS20-2189/2016**	93.1	93.1	93.1	92.9	93.1	92.8	93.1	93.1	93.0	92.8	93.1	93.1	93.1	93.1	92.4	92.5	92.4	92.5	92.3	92.4	96.9	96.5		
1D	**RusVs/yellow-necked field mouse/MV.DEU/KS20-2242/2013**	95.3	95.2	95.2	95.0	95.2	95.1	95.1	95.2	95.2	95.2	95.2	95.2	95.2	95.2	92.6	92.7	92.5	92.6	92.6	92.6	93.0	93.1	93.1	

Supplementary Figure S3: Nucleotide sequence identity matrix of an alignment of complete rustrela virus (RusV) genome sequences including 10 sequences established in this study. Sequences generated during this study are depicted in bold. RusV sequence names are shown in the format “host/ISO 1366 code of location (federal state.country)/animal ID/year”.

		KS20-1512	KS20-1535	KS21-0093	KS21-0096	KS21-0099	KS21-0101	KS21-0103	KS21-0106	KS21-0110	KS20-1592	KS20-1610	KS20-2915	KS20-2931	KS20-2266	KS20-2273	KS20-1409	KS20-1423	KS20-1655	KS20-1815	KS20-1852	KS20-2054
	**Lineage**	**1A**									**1B**						**1C**					
	**Sampling place**	**MV4**		**MV5**							**MV2**				**MV3**		**BB1**		**MV1**			
**CNS & spinal cord**	brain	19.4	20.9	-	-	26.9	-	-	-	-	26.4	27.9	26.5	26.7	28.3	24.3	22.2	23.4	29.2	25.4	25.9	25.6
**CNS & spinal cord**	cervical spinal cord	25.9	24.6	30.7	30.2	28.1	34.6	32.4	30.8	25.8	32.1	29.8	34.6	26.9	33.2	28.1	-	-	29.6	27.8	30.3	28.1
**CNS & spinal cord**	thoracal spinal cord	28.2	27.2	-	-	-	-	-	-	-	32.7	32.5	29.7	27.7	33.9	29.5	-	-	31.6	28.8	29.9	29.5
**CNS & spinal cord**	lumbal spinal cord	26.2	28.1	-	-	-	-	-	-	-	30.7	29.2	37.9	neg.	36.6	38.9	-	-	31.0	28.6	29.2	30.0
**eye & periphal nervs**	eye	27.7	34.7	-	-	36.7	-	-	-	-	33.7	36.5	neg.	29.6	neg.	35.0	-	-	29.4	31.3	-	33.0
**eye & periphal nervs**	Plexus brachialis	neg.	neg.	-	-	-	-	-	-	-	neg.	36.9	neg.	neg.	neg.	neg.	-	-	neg.	35.9	neg.	neg.
**eye & periphal nervs**	N. ischiadicus	38.4	neg.	39.6	37.2	39.3	neg.	neg.	neg.	neg.	neg.	neg.	neg.	36.4	neg.	neg.	-	-	neg.	38.7	neg.	neg.
**eye & periphal nervs**	adrenal glands	35.6	31.3	neg.	36.9	neg.	neg.	37.0	neg.	-	neg.	neg.	neg.	neg.	neg.	34.3	-	-	neg.	36.0	neg.	neg.
**head**	salivary gland	neg.	neg.	-	-	-	-	-	-	-	neg.	neg.	neg.	neg.	neg.	neg.	-	-	neg.	neg.	-	33.3
**head**	nasal septum	39.7	neg.	-	-	-	-	-	-	-	-	-	38.9	neg.	neg.	neg.	-	-	-	-	-	-
**thorax**	heart	neg.	neg.	neg.	neg.	neg.	neg.	neg.	neg.	neg.	neg.	neg.	neg.	neg.	neg.	neg.	neg.	neg.	35.3	neg.	neg.	neg.
**thorax**	lung	neg.	neg.	neg.	neg.	neg.	neg.	neg.	neg.	neg.	neg.	neg.	neg.	neg.	neg.	neg.	neg.	neg.	neg.	neg.	neg.	neg.
**thorax**	thoracic lavage	neg.	neg.	neg.	neg.	neg.	neg.	neg.	neg.	neg.	neg.	neg.	neg.	neg.	neg.	neg.	neg.	neg.	35.6	neg.	neg.	neg.
**abdomen**	spleen	neg.	neg.	neg.	neg.	neg.	neg.	neg.	neg.	neg.	neg.	neg.	neg.	neg.	neg.	neg.	neg.	neg.	neg.	neg.	neg.	neg.
**abdomen**	liver	neg.	neg.	neg.	neg.	neg.	neg.	neg.	neg.	neg.	neg.	neg.	neg.	neg.	neg.	neg.	neg.	neg.	neg.	neg.	neg.	neg.
**abdomen**	stomach	neg.	neg.	-	-	-	-	-	-	-	neg.	neg.	neg.	neg.	neg.	neg.	-	-	neg.	neg.	neg.	37.1
**abdomen**	small intestine	neg.	neg.	-	-	-	-	-	-	-	neg.	neg.	neg.	neg.	neg.	neg.	-	-	neg.	neg.	neg.	neg.
**abdomen**	colon	neg.	neg.	-	-	-	-	-	-	-	neg.	neg.	neg.	neg.	neg.	neg.	-	-	neg.	neg.	neg.	neg.
**abdomen**	kidney	neg.	neg.	neg.	neg.	neg.	neg.	neg.	neg.	neg.	neg.	neg.	neg.	neg.	neg.	neg.	neg.	neg.	neg.	neg.	neg.	neg.
**abdomen**	urinary bladder	neg.	neg.	-	-	-	-	-	-	-	-	-	neg.	neg.	neg.	neg.	-	-	-	-	-	-
**abdomen**	gonads/ embryos	34.0	neg.	-	-	-	-	-	-	-	neg.	neg.	neg.	neg.	neg.	neg.	-	-	neg.	neg.	neg.	neg.
**other**	skin	neg.	neg.	-	-	-	-	-	-	-	neg.	neg.	37.2	neg.	neg.	neg.	-	-	neg.	neg.	neg.	neg.
**other**	oral-swab	38.0	34.5	-	-	-	-	-	-	-	neg.	neg.	neg.	neg.	neg.	neg.	-	-	neg.	neg.	-	neg.

Supplementary Figure S4: **Distribution of RusV RNA in yellow-necked field mice.** A RusV-specific RT-qPCR (“Assay 1a”) was used to analyse the distribution of RusV RNA in different tissues from 21 selected yellow-necked field mice (*Apodemus flavicollis*). These yellow-necked field mice were detected to contain RNA of RusV lineages 1A-1C and originate from sites MV1-MV5 and BB1 (see **Fig. 1a**). The red color gradient highlights the abundance of RusV RNA detection, as represented by cycle of quantification (Cq) values. Samples represented by grey cells were not available for analysis.

Supplementary Table S1: Detection of RusV RNA in small mammals collected in the initially affected zoo and its surroundings. Presence of the virus in the tissues was (re-)assessed by RT-qPCR (“Assay 1a”).

	**Zoo**			**Within 2 km of zoo**			**Within 10 km of zoo**			**total**
	Bennett *et al., 2020a,b*	Pfaff *et al., 2022*	this study	Bennett *et al., 2020a,b*	Pfaff *et al., 2022*	this study	Bennett *et al., 2020a,b*	Pfaff *et al., 2022*	this study	
**Yellow-necked field mouse** *Apodemus flavicollis*	6/11	2/2	7/19	1/4	-	-	1/1	1/1	0/5	18/43
**Striped field mouse** *Apodemus agrarius*	0/4	-	-	-	-	-	0/2	-	0/1	0/7
**House mouse** *Mus musculus*	0/3*	-	-	-	-	-	0/13	-	-	0/16
**Norway rat** *Rattus norvegicus*	0/13*	-	0/3	-	-	-	-	-	-	0/16
**Bank vole** *Myodes glareolus*	0/3	-	0/2	-	-	-	-	-	-	0/5
**Common shrew** *Sorex araneus*	-	-	-	-	-	-	-	-	0/2	0/2

-, no material available.

*Two Norway rats and all three house mice were housed at the zoo.

Supplementary Table S2: **Sequences of primers and probes used for detection and sequencing of rustrela virus (RusV) RNA.** Primers and probes of RT-qPCR "Assay 1" were adjusted in order to reflect RusV diversity. Adjusted nucleotides in "Assay 1a" primer and probe sequences with respect to "Assay 1" are highlighted in bold.

Name	Sequence (5'→3')	Type	Usage
**RusV_1072+**	CGAGCGTGTCTACAAGTTCA	Primer	Assay 1
**RusV_1072_A+**	CGAGCG**Y**GTCTACAAGTT**Y**A	Primer	Assay 1a
**RusV_1237-**	GACCATGATGTTGGCGAGG	Primer	Assay 1/1a
**RusV_1116_P**	FAM-CCGAGGAGGACGCCCTGTGC-BHQ1	Probe	Assay 1
**RusV_1116_A_P**	FAM-CCGAGGA**R**GACGCCCTGTGC-BHQ1	Probe	Assay 1a
**RusV_323-**	TCGCCCCATTCWACCCAATT	Primer	5' RACE
**RusV_GSP1_M13**	CAGGAAACAGCTATGACCTATCTGCTCCTCGACCAACC	Primer	5' RACE
**M13_rev**	CAGGAAACAGCTATGACC	Primer	5' RACE
**AUAP**	GGCCACGCGTCGACTAGTAC	Primer	5' RACE
**AAP**	GGCCACGCGTCGACTAGTACGGGIIGGGIIGGGIIG	Primer	5' RACE
**AP**	GGCCACGCGTCGACTAGTACTTTTTTTTTTTTTTTTT	Primer	5' RACE
**RusV-E1_8188+**	CCRGTGGATGGCGGCTGCTT	Primer	E1 sequencing
**RusV-E1_8663-**	CTCGGGAGGCTRCACACG	Primer	E1 sequencing
**RusV-E1_8528+**	TCGCAGGGTAYGTCTATCAY	Primer	E1 sequencing
**RusV-E1_8941-**	CAATCACAGGCTGGTACCA	Primer	E1 sequencing

Supplementary Table S3: **High-throughput sequencing results for RNA pools of *Apodemus* brain samples.** The reads were screened for rubivirus specific sequences using diamond BLASTx search.

Sampling place (see Fig. 1a)	Pool ID	Number of animals in pool		Reads sequenced	Rubiviral BLASTx hits
		Apodemus flavicollis	*Apodemus sylvaticus*		
**TH**	lib04432	20	-	14,246,723	0
**HE**	lib04431	20	-	17,982,927	0
**NI**	lib04430	20	-	22,542,868	0
**BY-1**	lib04429	18	2	19,163,472	0
**BY-2**	lib04428	5	12	19,097,386	0
				93,033,376	0

TH, Thuringia; HE, Hesse; NI, Lower Saxony; BY, Bavaria

Supplementary Table S4: Results of the binomial generalized linear mixed effect model showing ecological risk factors associated with the infection risk of yellow-necked field mice. A PostHoc test was applied to the factor sampling site, as it was a categorical factor with 6 levels. Here all two-way comparisons between sites are presented. Sex, site-specific species richness and trapping year and season were eliminated during backwards model selection. Significant *p-values* < 0.05 are highlighted in bold.

Source of Variation	Estimate	Standard Error	z-value	p-value
**Intercept**	-4.984	0.937	-5.317	**<0.001**
**Mass**	0.093	0.023	4.023	**<0.001**
PostHoc on Site				
**MV5 vs. MV4**	0.597	0.689	0.866	0.954
**MV5 vs. MV3**	2.079	0.633	3.285	**0.013**
**MV5 vs. MV2**	2.104	0.755	2.789	0.058
**MV5 vs. MV1**	2.164	0.700	3.090	**0.024**
**MV5 vs. BB1**	2.255	0.732	3.081	**0.025**
**MV4 vs. MV3**	1.482	0.570	2.601	0.095
**MV4 vs. MV2**	1.507	0.711	2.119	0.274
**MV4 vs. MV1**	1.567	0.664	2.361	0.168
**MV4 vs. BB1**	1.658	0.673	2.466	0.132
**MV3 vs. MV2**	0.025	0.643	0.039	1.000
**MV3 vs. MV1**	0.085	0.594	0.143	1.000
**MV3 vs. BB1**	0.176	0.594	0.296	1.000
**MV2 vs. MV1**	0.060	0.729	0.082	1.000
**MV2 vs. BB1**	0.151	0.735	0.205	1.000
**MV1 vs. BB1**	0.091	0.694	0.131	1.000

References

1. Bennett AJ, Paskey AC, Ebinger A et al. (2020a) Relatives of rubella virus in diverse mammals. Nature 586:424–428. https://doi.org/10.1038/s41586-020-2812-9

2. Bennett AJ, Paskey AC, Ebinger A et al. (2020b) Author Correction: Relatives of rubella virus in diverse mammals. Nature 588:E2. https://doi.org/10.1038/s41586-020-2897-1

3. Pfaff F, Breithaupt A, Rubbenstroth D et al. (2022) Revisiting Rustrela Virus: New Cases of Encephalitis and a Solution to the Capsid Enigma. Microbiol Spectr 10:e0010322. https://doi.org/10.1128/spectrum.00103-22
